# The role of hepatocyte growth factor in the relationship between body fat distribution and plasma markers of glucose metabolism: a cross-sectional study

**DOI:** 10.1186/s13098-026-02096-1

**Published:** 2026-01-27

**Authors:** Katarina Zakic, Dennis Freuer, Jakob Linseisen, Christa Meisinger

**Affiliations:** 1 Epidemiology, Medical Faculty, University of Augsburg, Augsburg, Germany; 2 Institute of Epidemiology, University Hospital Augsburg, Stenglinstraße 2, Augsburg, 86156 Germany

**Keywords:** Hepatocyte growth factor, Visceral adipose tissue, Glucose metabolism, HbA1c, Bioelectrical impedance analysis

## Abstract

**Background:**

Obesity, particularly central adiposity, is a major risk factor for impaired glucose metabolism and type 2 diabetes. The biological mechanisms linking body fat distribution to glucose regulation remain incompletely understood. Therefore, we investigated the associations between body fat distribution and plasma markers of glucose metabolism in a population-based sample and examined the mediating role of hepatocyte growth factor (HGF) in this connection.

**Methods:**

The analysis was based on data from 238 participants of the MEGA study (German acronym for metabolic health study Augsburg) conducted between 2018 and 2021. Anthropometric measurements and a body composition analysis via bioelectrical impedance analysis (BIA) were conducted. HGF was measured from EDTA plasma based on the Proximity Extension Assay (Olink inflammation panel). Multivariable linear regression models were chosen to examine the associations between standardized anthropometric and BIA measurements and the outcome variables in non-diabetic individuals. Obesity measures included body mass index (BMI), waist circumference (WC), visceral adipose tissue (VAT), and waist-to-hip ratio (WHR), relative fat mass (RFMV), and absolute fat mass (AFMV). The outcome variables included fasting glucose, two-hour OGTT glucose, and HbA1c concentrations. Mediation effects of HGF between obesity measures and glucose parameters were assessed.

**Results:**

The observed positive associations between obesity measures and glucose metabolism were stronger for variables that are indicative of central obesity (WC, WHR, VAT), when compared to indices of general obesity (BMI, RFMV, AFMV). Fasting glucose showed the strongest positive association with WC (β = 5.10 mg/dL per one standard deviation increase; 95% CI 1.86–5.77). For two-hour plasma glucose, the strongest associations were observed with WHR (β = 21.00; 95% CI 14.57–27.44). HGF mediated between 7.0% and 9.1% of the total effects with two-hour glucose levels but not the associations with the other outcomes. No associations were found with HbA1c levels after accounting for multiple testing.

**Conclusions:**

These findings suggest HGF may contribute to the metabolic effects of different obesity measures on post-load glucose regulation, providing insights into obesity-related glucose dysregulation and potential targets for early intervention. However, the causal role of HGF remains unproven; further studies on the exact pathophysiological mechanisms are necessary.

**Supplementary Information:**

The online version contains supplementary material available at 10.1186/s13098-026-02096-1.

## Background

Diabetes is a global public health crisis, associated with high morbidity, mortality, and economic costs [1, 2]. In 2021, approximately 11% of adults aged 20–79 had diabetes, predominantly type 2 diabetes, and this number is rising rapidly [1]. Obesity is a leading risk factor for type 2 diabetes [2], and its prevalence has doubled in more than 70 countries since 1980, driven by unhealthy diets, physical inactivity, genetic predisposition, and environmental factors [3, 4]. Although body mass index (BMI) is commonly used to assess obesity, it does not accurately reflect body composition or the risks associated with obesity [5, 6]. Instead, central adiposity – particularly visceral fat accumulation – has been more strongly linked to metabolic disturbances, increased morbidity, and mortality [7–12]. Obesity increases diabetes risk primarily through chronic low-grade inflammation [13], with pro-inflammatory cytokines such as TNF-α and IL-6 acting as mediators between adiposity and whole-body insulin resistance, thereby contributing to the progression from prediabetes to overt type 2 diabetes [14]. Prediabetes encompasses two distinct conditions, namely impaired fasting glucose (IFG) and impaired glucose tolerance (IGT), each with unique underlying pathophysiology [15]. IFG mainly results from hepatic insulin resistance, which leads to increased hepatic glucose production and elevated fasting blood glucose, while insulin sensitivity in skeletal muscle is generally preserved. IFG is also characterized by reduced basal insulin release and a markedly weakened early-phase insulin secretion, whereas the later-phase response remains largely intact. In contrast, IGT arises primarily from insulin resistance in peripheral tissues, especially muscle, resulting in reduced glucose uptake in non-hepatic organs and consequently prolonged post-load hyperglycemia [15]. These differences underscore how IFG and IGT may represent distinct pathways in the progression towards type 2 diabetes.

Although obesity is strongly associated with impaired glucose metabolism and insulin resistance, the precise biological pathways connecting adiposity to metabolic dysfunction remain incompletely understood. This gap highlights the need to investigate additional biomarkers that may mediate the relationship between body fat distribution and glucose metabolism. One promising candidate is hepatocyte growth factor (HGF), a cytokine predominantly produced by adipose tissue, with serum levels positively associated with adiposity [16]. Previous research from our group has demonstrated strong associations between HGF and various anthropometric measures [17]. HGF plays multifaceted roles in metabolic regulation: it modulates glucose uptake and metabolism in various insulin-sensitive tissues (including pancreatic β-cells, enterocytes, adipocytes, hepatocytes, and skeletal muscle cells), supports β-cell homeostasis through proliferation, survival, and functional maintenance, and exerts anti-inflammatory effects that help to mitigate chronic inflammation often associated with metabolic disorders [18]. Given these properties, examining HGF as a potential mediator in the relationship between different obesity measures and glucose metabolism (such as impaired fasting glucose, impaired glucose tolerance, or HbA1c) could provide insights into whether HGF plays a mechanistic role in basal glucose homeostasis, glucose load handling, or long-term glycemia [18]. The present study therefore investigated associations between the obesity measures BMI, waist circumference (WC), visceral adipose tissue (VAT), waist-to-hip ratio (WHR), relative fat mass (RFMV), and absolute fat mass (AFMV) and plasma glucose markers (fasting glucose, two-hour OGTT (oral glucose tolerance test) glucose, HbA1c) in a population-based sample, with a particular focus on the potential mediating role of HGF in the adiposity–glucose metabolism axis.

## Materials and methods

The study was based on data from the MEGA study (German acronym for metabolic health study Augsburg), a population-based study conducted in Augsburg, Germany. A total of 238 men and women aged 25 to 65 years were interviewed and examined up to four times within a period of 9 months (baseline visit, follow-up visits after one and six months and a final visit after nine months) between 2018 and 2021. At the baseline visit, in addition to standardized questionnaires (e.g. information on lifestyle, sociodemographic data, information on previous illnesses assessed by a face-to-face interview) [19] anthropometric measurements, and a body impedance measurement were carried out in all participants. Furthermore, laboratory data, results of OGTTs, and long-term glucose measurements were available. However, at the follow-up visits, a reduced examination program with only a few selected tests was carried out. In particular, no OGTTs were performed at the follow-up visits. Therefore, this analysis was based on data from the baseline examination.

### Data collection

Education level was defined according to ISCED and categorized as low/medium (primary, lower secondary, upper secondary, post-secondary non-tertiary) and high (first- and second-stage tertiary) [20]. Blood pressure was measured three times with 2-minute intervals between the measurements at the right arm in a sitting position after a five-minute rest using a validated automatic device (OMRON M700 Intelli IT). The mean value of the second and third measurement was used for the analysis. Participants who were aware of having hypertension, who were taking antihypertensive medication, and/or who had systolic blood pressure of more than 140 mm Hg or diastolic blood pressure more than 90 mm Hg (with or without antihypertensive medications) were defined as having hypertension. Previously known diabetes was defined based on self-reported physician diagnosis or use of antidiabetic agents. To assess alcohol consumption patterns, the Alcohol Use Disorders Identification Test – Consumption (AUDIT-C) screening tool was used. Active alcohol use was defined as AUDIT-C score ≥ 3 for women or ≥ 4 for men [21]. Smoking status was categorized into “current smoker”, “former smoker”, and “never smoker. Physical activity (PA) was assessed using the Global Physical Activity Questionnaire (GPAQ) [22]. The time spent engaging in PA was transformed into metabolic equivalent (MET) time per week or MET min/week. Individuals who measured less than 600 MET, between 600 and 1500 MET, and more than 1500 MET min/week were classified as having low, moderate, and high levels of PA. Total serum cholesterol was measured using an enzymatic colorimetric method (CHOL2 assay; Cobas c analyzer, Roche Diagnostics GmbH, Mannheim, Germany). HDL (High-Density Lipoprotein) cholesterol and LDL (Low-Density Lipoprotein) cholesterol were measured using homogeneous enzymatic colorimetric assays (HDLC4 and LDLC3 assays, respectively; Cobas c analyzer, Roche Diagnostics GmbH, Mannheim, Germany). Triglycerides were also measured by an enzymatic colorimetric method (GPO-PAP), on a Cobas c analyzer (Roche Diagnostics GmbH, Mannheim, Germany).

Further information regarding the MEGA study can be found in a previous publication [23]. All examinations were performed in a fasted state following at least a 12-hour overnight fast and carried out by trained study nurses in accordance with previously defined standard operating procedures.

All study participants gave written informed consent. Methods of data and bio-sample collection have been approved by the ethics committee of the Ludwig-Maximilians Universität München and the study was performed in accordance with the Declaration of Helsinki. The study was registered at the DRKS (“Deutsches Register Klinischer Studien”) with the project number DRKS00015784.

### Exposure

Anthropometric measurements (height, weight, WC, hip circumference) and body composition analysis via bioelectrical impedance analysis (BIA, SECA mBCA515 device) were conducted. Body weight was measured to the nearest 0.1 kg wearing light clothing without shoes, and height was measured to the nearest 0.5 cm using a stadiometer. WC was measured with a tape measure at the narrowest point of the abdomen, and hip circumference was measured to the nearest 0.1 cm at the maximum bulge of the hips at the level of the pubic symphysis. BMI was calculated as weight in kilograms divided by the square of height in meters. For the SECA assessment subjects stood barefoot and dressed in shorts and T-shirts on the platform of the device and grasped the grip rod as appropriate for their height. Bioelectrical impedance is measured using an 8-point electrode configuration (four pairs: two on each hand via handrail grips and two on each foot via platform electrodes). The device measures raw impedance data (resistance, reactance, phase angle) across body segments, particularly focusing on the torso where visceral fat is located. From body weight and bioelectrical impedance measurements, the device calculates and reports key body composition parameters such as fat mass, fat free mass, skeletal muscle mass, and visceral fat mass. Body composition parameters are calculated using proprietary, clinically validated algorithms developed by SECA. These equations incorporate raw impedance data, weight, height (manually entered or transmitted wirelessly), age, sex, and ethnicity. For the present analysis, the following parameters were used as exposures: BMI (kg/m^2^), WC (cm), WHR, VAT (L), RFMV (%), and AFMV (kg).

### Measurement of HGF

HGF was measured as part of the determination of 92 protein markers from EDTA plasma using the Olink inflammation panel. The measurement is based on the Proximity Extension Assay (PEA). Information on this process can be found at the website of Olink Proteomics (https://olink.com/technology) or in a prior publication [17].

### Outcome

The OGTT was performed in the morning (7:00 to 11:00 a.m.). Participants were asked to fast for 12 h overnight, to avoid strenuous physical activity on the day before the examination, and not to smoke before or during the test. Exclusion criteria for the OGTT were taking antidiabetic medications or known diabetes, and acute illnesses (infection, fever, acute gastrointestinal disease). The following glucose measurements were determined as outcomes: fasting plasma glucose, two-hour plasma glucose after ingestion of 75 g glucose in the framework of the OGTT in non-diabetic participants, and the HbA1c value. For the blood collection, NaF/citrate plasma tubes (GlucoEXACT) and EDTA plasma tubes were used, and the measurements were performed at the laboratory of the University Hospital Augsburg immediately after collection. HbA1c was measured by a reverse-phase cation-exchange high-pressure liquid chromatography (HPLC, Analyzer HA 8160; Menarini, Florence, Italy).

### Statistical analysis

Depending on their distributions, continuous variables were presented either as mean and standard deviation (SD) or as median and interquartile range (IQR). Normally distributed variables were analyzed using t-tests; otherwise, Mann-Whitney-U tests were applied. Categorical variables were presented as absolute frequencies as well as column percentages and analyzed using Chi-square tests.

Multivariable linear regression models were chosen to examine the associations between the exposure variables (VAT, AFMV, RVMV, BMI, WC, and WHR) and outcome variables (fasting glucose, two-hour OGTT glucose, and HbA1c levels). To ensure comparability of the effect sizes, the exposure variables were standardized (i.e. $\:\frac{X-\mu\:}{\sigma\:}$, so that $\:X\sim N\left(\mathrm{0,1}\right)$). All the assumptions of linear regression were checked and ensured. Normal distribution of the regression residuals was checked graphically. Potential outliers were investigated and identified using Cook´s distance.

Potential confounders were assessed and selected using a directed acyclic graph (DAG) (Supplementary Figure S2). Consequently, all models were adjusted for the following variables: age (years), sex, smoking status (current smoker, former smoker, never smoker), alcohol consumption (defined as AUDIT-C-Score ≥ 3 for women or ≥ 4 for men), physical activity (low, moderate, high) and education (low/medium, high) [24]. Information on potential further confounders (diet quality, specific medications) was not available possibly introducing bias. Furthermore, the cross-sectional design inherently limits causal inference. Effect modification of age and sex was assessed by testing the respective interaction terms.

Mediation effects of HGF were assessed by modelling and testing the total effects (i.e. exposure-outcome associations without the mediator), direct effects (i.e. exposure-outcome associations considering the mediator), and indirect effects, i.e. the exposure-mediator as well as mediator-outcome associations using multivariable linear regression models. The indirect effects (path from exposure to outcome over mediator) were assessed using bootstrapping for confidence intervals (CIs). Additionally, interactions between exposures and the mediator were tested. After decomposing the individual paths between exposure, mediator, and outcome, the proportion mediated was calculated (where appropriate) by dividing the point estimate of the indirect effect by the point estimate of the total effect (i.e. $\:\frac{{\beta\:}_{indirect}}{{\beta\:}_{total}}$) [25].

After excluding participants with diabetes, 228 individuals were considered in the regression analyses (Supplementary Figure S1). Since the proportion of missing values was low (Supplementary Figure S1) and the mechanism of missing values could be considered completely random, a complete case analysis was performed. The reported effect estimates (β-coefficient and 95% CI) represent the expected change in the respective outcomes associated with one standard deviation (SD) increase in the exposure variable. Regarding the 18 null-hypotheses of the research question (6 exposures and 3 outcomes), results with P-values below the Bonferroni-corrected threshold of 0.003 were considered statistically significant. All statistical analyses were performed using SPSS (version 29.0). The PROCESS macro for SPSS provided by A. F. Hayes was used for the mediation analysis [26].

## Results

Table 1 presents the characteristics for the total sample (*N* = 238 individuals) and stratified by sex. About two-thirds of all study participants were women, and the median age was 48 (IQR 36–56) years. Nearly 4.3% of all study participants had diabetes, less than half reported a low to moderate level of physical activity, and about two-thirds of the sample had a low to medium level of education.

On average, men had a higher BMI and more visceral fat mass than women. While the median HbA1c and median two-hour OGTT glucose values did not differ significantly between the sexes, men had higher fasting glucose values. Men were more likely to have hypertension and more likely to be current or former smokers compared to women.

### Total effects

After multivariable adjustment, all exposures (VAT, AFMV, RFMV, BMI, WC and WHR) were positively associated with fasting plasma glucose concentrations as well as the two-hour plasma glucose levels (*P* < 0.001) (Table 2). However, considering the Bonferroni-adjusted significance threshold of 0.003, none of the exposures were associated with HbA1c levels (Table 2). Tests for interactions showed no evidence that age or sex modified any association.

Basically, the associations between body fat distribution and two-hour plasma glucose levels, which yielded point estimates between 13.76 and 21.00 mg/dL per one SD increase of the respective exposure variables, were stronger than the estimates for fasting plasma glucose concentrations between 3.82 and 5.10 mg/dL (Table 2); this finding suggests that two-hour glucose might be a more sensitive indicator of metabolic health than fasting glucose or HbA1c. Furthermore, estimates quantifying associations with visceral fat content (i.e. VAT, WC, and WHR) were (except for the WHR-fasting glucose association) consistently larger than the estimates regarding the total body fat content (i.e. AFMV, RFMV, and BMI), highlighting the importance of central adiposity.

### Mediation analyses

Mediation analyses revealed that HGF acted as a partial mediator in the associations between body fat distribution and two-hour plasma glucose levels (Supplementary Table S3). The proportion of mediation by HGF was between 7.0% and 9.1% of the total effect with RFMV and AFMV, respectively (Table 3). No mediating effects were observed in the associations with fasting plasma glucose or HbA1c levels (Table 3, Supplementary Table S3). This was particularly due to the lack of associations between HGF and fasting plasma glucose as well as HbA1c concentrations (despite a strong association between body fat distribution and the mediator) (Supplementary Table S3).

After accounting for the association-specific mediation effects of HGF, all parameters describing the body fat distribution remained strongly associated (in terms of direct effects) with the respective outcomes (Table 3). There was no evidence of any exposure-mediation interaction (Supplementary Table S3).

**Table 1:** Baseline characteristics of the overall study sample

	Number of participants (Women, Men)	Total study sample	Women	Men	*P*-value
Age, y	238 (164, 74)	48 (36, 56)	47.5 (38, 54)	49 (35, 59)	0.302
BMI, kg/m2	238 (164, 74)	25.5 (22.7, 33.1)	24.5 (21.9, 33)	30.5 (24.5, 34.1)	0.001
WC, cm	238 (164, 74)	88.5 (77, 107)	84 (74, 100.7)	104.5 (85, 119.2)	< 0.001
WHR	237 (163, 74)	0.84 (0.77, 0.93)	0.81 (0.74, 0.87)	0.98 (0.86, 1)	< 0.001
VAT, L	227 (156, 71)	1.4 (0.78, 3.41)	1.17 (0.58, 2.19)	3.8 (1.19, 6.51)	< 0.001
FMI, kg/m2	227 (156, 71)	8.6 (5.9, 13.5)	8.6 (6.4, 15)	8.44 (4.23, 12)	0.039
AFMV, kg	227 (156, 71)	24.4 (17, 40.1)	27.3 (17.6, 40.3)	27.1 (13.7, 40)	0.527
RFMV, * % body weight	227 (156, 71)	34 ± 10.5	36.8 ± 9.3	27.6 ± 10.1	< 0.001
Fasting plasma glucose, mg/dL	225 (156, 69)	97 (92.5, 104)	96 (90.5, 102)	101 (96, 108.5)	< 0.001
Two-hour oral glucose tolerance test, mg/dL	212 (144, 68)	106.5 (87, 127.5)	103.5 (86.2, 125.7)	107.5 (88.2, 132.5)	0.619
HbA1c, mmol/mol	234 (161, 73)	35 (32, 37)	35 (32, 37)	36 (32, 38.5)	0.192
Total cholesterol, * mg/dL	235 (161, 74)	186.5 ± 35.6	188.2 ± 33.5	182.9 ± 39.8	0.294
LDL cholesterol, * mg/dL	235 (161, 74)	120.5 ± 31.4	119.3 ± 30.6	123.1 ± 33.2	0.404
HDL cholesterol, mg/dL	235 (161, 74)	60 (48, 72)	65 (54, 77)	49.5 (42.75, 56.5)	< 0.001
Triglycerides, mg/dL	235 (161, 74)	82 (59, 124)	76 (57.5, 112)	110 (74.7, 157.5)	< 0.001
Hypertension	235 (161, 74)	63 (26.81%)	32 (19.88%)	31 (41.89%)	< 0.001
Diabetes	235 (162, 73)	10 (4.26%)	5 (3.09%)	5 (6.85%)	0.186
Smoking	238 (164, 74)				0.011
Current		34 (14.29%)	23 (14.02%)	11 (14.86%)	
Former		85 (35.71%)	49 (29.88%)	36 (48.65%)	
Never		119 (50.00%)	92 (56.10%)	27 (36.49%)	
Active alcohol use °	238 (164, 74)	116 (48.70%)	78 (47.60%)	38 (51.40%)	0.588
Physical activity	238 (164, 74)				0.047
Low (< 600 MET/week)		36 (15.19%)	28 (17.07%)	8 (10.96%)	
Moderate (600–1500 MET/week)		67 (28.27%)	52 (31.71%)	15 (20.55%)	
High (> 1500 MET/week)		134 (56.54%)	84 (51.22%)	50 (68.49%)	
Education**	238 (164, 74)				0.720
Low/Medium		166 (69.75%)	113 (68.90%)	53 (71.62%)	
High		72 (30.25%)	51 (31.10%)	21 (28.38%)	

Abbreviations: AFMV, absolute fat mass; BMI, body mass index; FMI, fat mass index; RFMV, relative fat mass; VAT, visceral adipose tissue;

WC, waist circumference; WHR, waist-to-hip-ratio

* Normally distributed data are presented as mean ± SD

Non-normally distributed data are presented as median (25th, 75th percentiles)

**Defined as ISCED low/medium (primary, lower secondary, upper secondary, post-secondary non-tertiary) and high (first- and second-stage tertiary [20]

° Defined as AUDIT-C-Score ≥ 3 for women or ≥ 4 for men

**Table 2:** Associations (total effect estimates) between measures of body fat and plasma markers of glucose metabolism in non-diabetic participants (*n* = 228)

	β	95% Confidence Interval		*P*
		Lower	Upper	
Fasting plasma glucose levels (mg/dL)				
VAT	4.95	3.20	6.69	< 0.001
AFMV	4.21	2.77	5.64	< 0.001
RFMV	4.18	2.59	5.78	< 0.001
BMI	4.73	3.30	6.17	< 0.001
WC	5.10	3.52	6.67	< 0.001
WHR	3.82	1.86	5.77	< 0.001
Two-hour plasma glucose levels (mg/dL)				
VAT	17.80	12.02	23.57	< 0.001
AFMV	13.76	8.83	18.69	< 0.001
RFMV	16.47	11.21	21.72	< 0.001
BMI	15.83	10.90	20.76	< 0.001
WC	18.00	12.69	23.31	< 0.001
WHR	21.00	14.57	27.44	< 0.001
HbA1c levels (mmol/mol)				
VAT	0.92	0.21	1.63	0.011
AFMV	0.64	0.05	1.22	0.033
RFMV	0.77	0.13	1.41	0.018
BMI	0.75	0.15	1.35	0.014
WC	0.81	0.15	1.46	0.016
WHR	0.83	0.07	1.60	0.033

The linear models were adjusted for age. sex. smoking status. alcohol consumption. physical activity and education. Estimates can be interpreted as the expected change in an outcome, when increasing a respective exposure variable by one standard deviation. P-values below a Bonferroni-corrected threshold of 0.003 were considered statistically significant

Abbreviations: AFMV. absolute fat mass; BMI. body mass index; RFMV. relative fat mass; VAT. visceral adipose tissue; WC. waist circumference; WHR. waist-to-hip-ratio

**Table 3:** Direct effect estimates (i.e. exposure-outcome associations accounted for HGF) and the proportion mediated by HGF from mediation analyses between body fat distribution and plasma markers of glucose metabolism in non-diabetic participants (*n* = 228)

	β	95% Confidence Interval		*P*	Mediation (%)
		Lower	Upper		
Fasting plasma glucose levels (mg/dL)					
VAT	4.93	3.15	6.72	< 0.001	no
AFMV	4.18	2.72	5.64	< 0.001	no
RFMV	4.13	2.52	5.75	< 0.001	no
BMI	4.76	3.28	6.23	< 0.001	no
WC	5.14	3.52	6.77	< 0.001	no
WHR	3.75	1.74	5.76	< 0.001	no
Two-hour plasma glucose levels (mg/dL)					
VAT	16.32	10.53	22.11	< 0.001	8.3
AFMV	12.50	7.57	17.44	< 0.001	9.1
RFMV	15.32	10.10	20.53	< 0.001	7.0
BMI	14.46	9.46	19.46	< 0.001	8.6
WC	16.54	11.14	21.94	< 0.001	8.1
WHR	19.29	12.78	25.80	< 0.001	8.1
HbA1c levels (mmol/mol)					
VAT	0.95	0.22	1.67	0.011	no
AFMV	0.65	0.05	1.24	0.034	no
RFMV	0.78	0.13	1.43	0.018	no
BMI	0.77	0.16	1.38	0.014	no
WC	0.83	0.16	1.51	0.015	no
WHR	0.87	0.08	1.65	0.032	no

The linear models were adjusted for age. sex. smoking status. alcohol consumption. physical activity and education. Estimates can be interpreted as the expected change in an outcome, when increasing a respective exposure variable by one standard deviation. P-values below a Bonferroni-corrected threshold of 0.003 were considered statistically significant

Abbreviations: AFMV. absolute fat mass; BMI. body mass index; RFMV. relative fat mass; VAT. visceral adipose tissue; WC. waist circumference; WHR. waist-to-hip-ratio

## Discussion

In the present study, the associations between conventional body composition measurements as well as BIA measurements and plasma markers of glucose metabolism in a non-diabetic population-based sample were investigated, with a particular focus on the mediating role of HGF. It was found that all obesity indices were positively associated with fasting glucose and two-hour OGTT glucose but not with HbA1c levels. Generally, the associations were stronger for variables indicative of central obesity (VAT, WC, and WHR) compared to indices of general obesity (BMI, AFMV, or RFMV). HGF partially mediated the associations between BIA or anthropometric measurements and two-hour OGTT glucose levels, but not the associations with fasting glucose or HbA1c levels.

The present results suggest that HGF may be associated with acute post-load glucose fluctuation but not with basal glucose regulation. Previous studies have reported significant associations between HGF and glucose metabolism, although direct post-load glucose research is limited. For example, Garcia-Ocana et al. found that HGF overexpression in mice leads to mild hypoglycemia and increased insulin production [27]. Another study demonstrated a significant positive association between HGF and serum glucose levels in hypertensive subjects [28]. In a prospective study from the Multi-Ethnic Study of Atherosclerosis (MESA), a one standard deviation increase in HGF was associated with a 21% increased risk of diabetes [29]. Collectively, these findings suggest that HGF may exert beneficial metabolic effects at the tissue level, while elevated circulating concentrations may also reflect underlying metabolic stress or compensatory failure in obesity. Other research has shown that HGF regulates metabolism by stimulating hepatic glucose uptake and suppressing hepatic glucose release [30]. Weight loss has been found to improve hepatic insulin resistance by normalizing fasting glucose metabolism and reversing obesity-induced activation of HGF/HGF receptor c-Met pathway [31, 32]. The HGF/c-Met pathway (where HGF is hepatocyte growth factor and c-Met is its receptor) is a critical receptor tyrosine kinase signaling system that regulates cellular proliferation, migration, invasion, and survival through complex molecular interactions. Activation of this pathway initiates a cascade of intracellular signaling events, including ERK1/2, MAPK, STAT3, Rac1, and PI3K/AKT [33], which can profoundly influence cellular behavior [34].

The two-hour OGTT glucose primarily reflects the body’s ability to clear a glucose challenge, involving acute insulin secretion (first- and second-phase response from pancreatic β-cells), peripheral glucose uptake (especially in skeletal muscle and adipose tissue), and suppression of hepatic glucose output. HGF is known to promote β-cell proliferation, survival, and insulin secretion in response to metabolic stress such as obesity-induced insulin resistance. It also directly stimulates glucose uptake and metabolism in insulin-sensitive tissues, for example via PI3K/Akt pathways in myocytes and adipocytes [18, 33]. Mediation specifically through the two-hour OGTT pathway may indicate that obesity-elevated HGF is associated with post-load hyperglycemia, potentially involving β-cell adaptation or acute peripheral glucose handling during a glucose surge.

Fasting glucose is more heavily regulated by hepatic gluconeogenesis and basal insulin action in the liver. Although existing evidence shows that HGF can suppress hepatic glucose output and improve liver insulin signaling in some models [30, 32], the absence of mediation in the present study suggests that the link between obesity and fasting glycemia may operate through other dominant pathways (e.g., free fatty acids, chronic inflammation via TNF-α/IL-6, or direct hepatic lipotoxicity) [35–37].

Several studies have consistently shown associations between different obesity measurements and HbA1c. For example, a cross-sectional study of 99 non-diabetic young adults found a significant positive association between obesity measures (BMI, WC, body fat percentage) and HbA1c levels [38]. Another cross-sectional study based on NHANES data including 11,125 non-diabetic individuals demonstrated that total and trunk body fat were strongly associated with elevated HbA1c, with odds ratios ranging from 1.60 to 2.01 across age and sex categories [39]. In contrast, no associations between obesity measures and HbA1c were found in the present study after correction for multiple testing. This may be due to the fact that HbA1c reflects long-term average glycemia, which could dilute subtle associations in a non-diabetic, relatively healthy cohort.

Multiple prior studies provided robust evidence for the link between body fat distribution and glucose metabolism in non-diabetic individuals [40–43], which was supported by the present findings. We found higher beta coefficients for the association between body fat measures and two-hour plasma glucose than for the associations with fasting glucose and HbA1c. This finding could possibly be attributed to the fact that the two-hour plasma glucose level allows for a more comprehensive and dynamic assessment of metabolic function compared to the fasting glucose level and shows stronger relationships with body fat and insulin resistance. A prior study found a strong inverse relationship between total glucose disposal and two-hour plasma glucose concentration, which varied significantly across different glucose tolerance groups [44].

Another study demonstrated that increasing two-hour glucose levels are associated with progressive impairments in beta-cell responsiveness, independent of insulin resistance [45].

These findings were further supported by Weigensberg et al. showing that two-hour insulin values maintained significant independent associations with insulin sensitivity even after adjusting for body composition, whereas fasting measures did not [46].

## Clinical and research implications

The present finding would highlight HGF (or c-Met pathway activity) as a potential biomarker or target specifically for impaired glucose tolerance (elevated two-hour OGTT) rather than isolated impaired fasting glucose. However, these aspects are clearly hypothetical, particularly since c-Met signaling is also involved in oncogenic pathways [47]. If future research will confirm that HGF mediates the obesity–glucose metabolism relationship, it could serve as a biomarker for early metabolic dysfunction and help to identify individuals at higher risk for progressing from obesity to impaired glucose tolerance or type 2 diabetes, enabling personalized interventions. In addition, there seems to be mechanistic differences: obesity-to-dysglycemia pathways may diverge, with post-load defects more linked to β-cell/peripheral compensation (involving HGF) and fasting defects more hepatic/lipotoxicity-driven [15, 18]. Overall, this selective mediation is consistent with an association between HGF and post-load glucose regulation, although whether this response is ultimately protective or maladaptive cannot be determined from cross-sectional data.

Future research should explore whether circulating HGF can serve as a biomarker for early stages of post-load glucose dysregulation. Based on studies using dynamic tests (e.g., mixed-meal tolerance) the specificity of HGF to glucose challenges could be confirmed. If it is confirmed that HGF plays a role in post-load hyperglycemia associated with obesity, whether as a compensatory or stress marker, this could lead to ideas for therapeutic options for prediabetes and early type 2 diabetes.

This study has several strengths. First, a relatively large number of well-phenotyped individuals was examined, and the measurements were all highly standardized. Furthermore, a wide range of data and information was collected for each participant, enabling adjustment for all relevant confounders. The study participants were primarily middle-aged, non-diabetic, and healthy, allowing assessment of relationships between fat compartments and risk factors in the absence of significant comorbidity. The present study also has limitations. Due to the cross-sectional design, reverse causality cannot be ruled out, even though the causal chain is fundamentally understood. Since the majority of study participants represent a typical German population aged 25–65 years, the results may not be generalized to other ethnicities and age-groups. Furthermore, the use of VAT estimated by BIA instead of computed tomography measurements (the gold standard) may have led to less accurate results, so measurement errors cannot be excluded. In the present study BIA-derived VAT did not outperform conventional measures (WC, WHR). Possible explanations for this could be that BIA is sensitive to factors such as hydration status, recent physical activity, mealtimes, and ethnicity, leading to fluctuations and reduced accuracy in measuring visceral fat percentage or central fat percentage [48]. In addition, no measures of insulin sensitivity were available in the present study. In the MEGA follow-up examinations, a reduced program with a few selected assessments was implemented; thus, no longitudinal analyses could be carried out to exclude reverse causality.

## Conclusions

The positive associations between measures of obesity and plasma markers of glucose metabolism were stronger for indices of visceral obesity than for indices of general obesity. However, the parameters determined by BIA measurement did not outperform conventional measurements in terms of their associations with the evaluated plasma markers of glucose metabolism. HGF is partially involved in these relationships, particularly in the association between different obesity measures and two-hour OGTT glucose values. Because mediation does not prove causality, further studies on the exact pathophysiological mechanisms are necessary.

## Supplementary Information

Supplementary Material 1

## Data Availability

The data underlying this article cannot be shared publicly because the data are subject to national data protection laws and restrictions that were imposed by the ethics committee of the Ludwig-Maximilians University, Munich, to ensure data privacy of the study participants because they did not explicitly consent to the data being made publicly available. The data will be shared at reasonable requests to the corresponding author.

## Abbreviations

AFMV: Absolute fat mass
AUDIT-C: Alcohol use disorders identification test - consumption
BIA: Bioelectrical impedance analysis
BMI: Body mass index
CI: Confidence interval
DAG: Directed acyclic graph
FMI: Fat mass index
HDL: High-Density Lipoprotein
HGF: Hepatocyte growth factor
IQR: Interquartile range
LDL: Low-Density Lipoprotein
MEGA: German acronym for metabolic health study Augsburg
MET: Metabolic equivalent
OGTT: Oral glucose tolerance test
RFMV: Relative fat mass
SAT: Subcutaneous adipose tissue
SD: Standard deviation
VAT: Visceral adipose tissue
WC: Waist circumference
WHR: Waist-to-hip-ratio

## References

[1] Lehner CT, Eberl M, Donnachie E, Tanaka LF, Schauberger G, Schederecker F, et al. Incidence trend of type 2 diabetes from 2012 to 2021 in Germany: an analysis of health claims data of 11 million statutorily insured people. Diabetologia. 2024;67:1040–50. 10.1007/s00125-024-06113-8. 38409438 10.1007/s00125-024-06113-8 PMC11058936

[2] Kang PS, Neeland IJ. Body fat distribution, diabetes mellitus, and cardiovascular disease: an update. Curr Cardiol Rep. 2023;25:1555–64. 10.1007/s11886-023-01969-5. 37792133 10.1007/s11886-023-01969-5

[3] Afshin A, Forouzanfar MH, Reitsma MB, Sur P, Estep K, Lee A, et al. Health effects of overweight and obesity in 195 countries over 25 years. N Engl J Med. 2017;377:13–27. 10.1056/nejmoa1614362. 28604169 10.1056/NEJMoa1614362 PMC5477817

[4] Catalán V, Avilés-Olmos I, Rodríguez A, Becerril S, Fernández-Formoso JA, Kiortsis D, et al. Time to consider the “Exposome Hypothesis” in the development of the obesity pandemic. Nutrients. 2022. 10.3390/nu14081597. 35458158 10.3390/nu14081597 PMC9032727

[5] Blundell JE, Dulloo AG, Salvador J, Frühbeck G. Beyond BMI - phenotyping the obesities. Obes Facts. 2014;7:322–8. 10.1159/000368783. 25485991 10.1159/000368783 PMC5644899

[6] Okorodudu DO, Jumean MF, Montori VM, Romero-Corral A, Somers VK, Erwin PJ, Lopez-Jimenez F. Diagnostic performance of body mass index to identify obesity as defined by body adiposity: a systematic review and meta-analysis. Int J Obes. 2010;34:791–9. 10.1038/ijo.2010.5. 10.1038/ijo.2010.5 20125098

[7] Faria AN, Filho FFR, Ferreira SRG, Zanella MT. Impact of visceral fat on blood pressure and insulin sensitivity in hypertensive obese women. Obes Res. 2002;10:1203–6. 10.1038/oby.2002.164. 12490663 10.1038/oby.2002.164

[8] Oppert J-M, Charles M-A, Thibult N, Guy-Grand B, Eschwège E, Ducimetière P. Anthropometric estimates of muscle and fat mass in relation to cardiac and cancer mortality in men: the Paris prospective study. Am J Clin Nutr. 2002;75:1107–13. 10.1093/ajcn/75.6.1107. 12036820 10.1093/ajcn/75.6.1107

[9] Bosy-Westphal A, Braun W, Geisler C, Norman K, Müller MJ. Body composition and cardiometabolic health: the need for novel concepts. Eur J Clin Nutr. 2018;72:638–44. 10.1038/s41430-018-0158-2. 29748654 10.1038/s41430-018-0158-2

[10] Zhu S, Wang Z, Heshka S, Heo M, Faith MS, Heymsfield SB. Waist circumference and obesity-associated risk factors among Whites in the third National health and nutrition examination survey: clinical action thresholds. Am J Clin Nutr. 2002;76:743–9. 10.1093/ajcn/76.4.743. 12324286 10.1093/ajcn/76.4.743

[11] Fox CS, Massaro JM, Hoffmann U, Pou KM, Maurovich-Horvat P, Liu C-Y, et al. Abdominal Visceral and Subcutaneous Adipose Tissue Compartments. Circulation. 2007;116:39–48. 10.1161/CIRCULATIONAHA.106.675355. 17576866 10.1161/CIRCULATIONAHA.106.675355

[12] Nicklas BJ. Association of visceral adipose tissue with incident myocardial infarction in older men and women: the Health, aging and body composition study. Am J Epidemiol. 2004;160:741–9. 10.1093/aje/kwh281. 15466496 10.1093/aje/kwh281

[13] Zatterale F, Longo M, Naderi J, Raciti GA, Desiderio A, Miele C, Beguinot F. Chronic adipose tissue inflammation linking obesity to insulin resistance and type 2 diabetes. Front Physiol. 2020. 10.3389/fphys.2019.01607. 32063863 10.3389/fphys.2019.01607 PMC7000657

[14] Bastard J-P, Maachi M, Lagathu C, Kim MJ, Caron M, Vidal H, et al. Recent advances in the relationship between obesity, inflammation, and insulin resistance. Eur Cytokine Netw. 2006;17:4–12. 16613757

[15] Abdul-Ghani M, DeFronzo RA, Jayyousi A. Prediabetes and risk of diabetes and associated complications: impaired fasting glucose versus impaired glucose tolerance: does it matter? Curr Opin Clin Nutr Metab Care. 2016;19:394–9. 10.1097/MCO.0000000000000307. 27389083 10.1097/MCO.0000000000000307

[16] Rehman J, Considine RV, Bovenkerk JE, Li J, Slavens CA, Jones RM, et al. Obesity is associated with increased levels of circulating hepatocyte growth factor. J Am Coll Cardiol. 2003;41:1408–13. 10.1016/S0735-1097(03)00231-6. 12706940 10.1016/s0735-1097(03)00231-6

[17] Schmitz T, Freuer D, Meisinger C, Linseisen J. Associations between anthropometric parameters and immune-phenotypical characteristics of circulating Tregs and serum cytokines. Inflamm Res. 2023;72:1789–98. 10.1007/s00011-023-01777-1. 37659013 10.1007/s00011-023-01777-1 PMC10539435

[18] Oliveira AG, Araújo TG, Carvalho B, Rocha GZ, Santos A, Saad MJA. The role of hepatocyte growth factor (HGF) in insulin resistance and diabetes. Front Endocrinol. 2018. 10.3389/fendo.2018.00503. 10.3389/fendo.2018.00503 PMC6125308 30214428

[19] Peters A, Greiser KH, Göttlicher S, Ahrens W, Albrecht M, Bamberg F, et al. Framework and baseline examination of the German National Cohort (NAKO). Eur J Epidemiol. 2022;37:1107–24. 10.1007/s10654-022-00890-5. 36260190 10.1007/s10654-022-00890-5 PMC9581448

[20] Data for the Sustainable Development Goals | Institute for Statistics (UIS). 22.12.2025. https://www.uis.unesco.org/en. Accessed 22 Dec 2025.

[21] Aalto M, Alho H, Halme JT, Seppä K. AUDIT and its abbreviated versions in detecting heavy and binge drinking in a general population survey. Drug Alcohol Depend. 2009;103:25–9. 10.1016/j.drugalcdep.2009.02.013. 19395203 10.1016/j.drugalcdep.2009.02.013

[22] Bull FC, Maslin TS, Armstrong T. Global physical activity questionnaire (GPAQ): nine country reliability and validity study. J Phys Act Health. 2009;6:790–804. 10.1123/jpah.6.6.790. 20101923 10.1123/jpah.6.6.790

[23] Schmitz T, Freuer D, Linseisen J, Meisinger C. Associations between serum cholesterol and immunophenotypical characteristics of circulatory B cells and Tregs. J Lipid Res. 2023;64:100399. 10.1016/j.jlr.2023.100399. 37276940 10.1016/j.jlr.2023.100399 PMC10394386

[24] Meisinger C, Döring A, Thorand B, Heier M, Löwel H. Body fat distribution and risk of type 2 diabetes in the general population: are there differences between men and women? The MONICA/KORA Augsburg cohort study. Am J Clin Nutr. 2006;84:483–9. 10.1093/ajcn/84.3.483. 16960160 10.1093/ajcn/84.3.483

[25] Tönnies T, Schlesinger S, Lang A, Kuss O. Mediation analysis in medical research. Dtsch Arztebl Int. 2023;120:681–7. 10.3238/arztebl.m2023.0175. 37584228 10.3238/arztebl.m2023.0175 PMC10666259

[26] Hayes AF, editor. Introduction to mediation, moderation, and conditional process analysis: A regression-based approach. New York, London: The Guilford Press; 2022.

[27] Garcia-Ocaña A, Takane KK, Syed MA, Philbrick WM, Vasavada RC, Stewart AF. Hepatocyte growth factor overexpression in the islet of transgenic mice increases beta cell proliferation, enhances islet mass, and induces mild hypoglycemia. J Biol Chem. 2000;275:1226–32. 10.1074/jbc.275.2.1226. 10625667 10.1074/jbc.275.2.1226

[28] Prlic MF, Brinar IV, Ivandic E, Knezevic T, Ivkovic V, Jelakovic B. #6873 hepatocyte growth factor correlates with serum glucose levels in hypertensive subjects. Nephrol Dial Transplant. 2023. 10.1093/ndt/gfad063c_6873.

[29] Bancks MP, Bielinski SJ, Decker PA, Hanson NQ, Larson NB, Sicotte H, et al. Circulating level of hepatocyte growth factor predicts incidence of type 2 diabetes mellitus: the Multi-Ethnic Study of Atherosclerosis (MESA). Metabolism. 2016;65:64–72. 10.1016/j.metabol.2015.10.023. 26892517 10.1016/j.metabol.2015.10.023 PMC4857763

[30] Fafalios A, Ma J, Tan X, Stoops J, Luo J, DeFrances MC, et al. A hepatocyte growth factor receptor (Met) − insulin receptor hybrid governs hepatic glucose metabolism. Nat Med. 2011;17:1577–84. 10.1038/nm.2531. 22081023 10.1038/nm.2531 PMC3233634

[31] Petersen KF, Dufour S, Befroy D, Lehrke M, Hendler RE, Shulman GI. Reversal of nonalcoholic hepatic steatosis, hepatic insulin resistance, and hyperglycemia by moderate weight reduction in patients with type 2 diabetes. Diabetes. 2005;54:603–8. 10.2337/diabetes.54.3.603. 15734833 10.2337/diabetes.54.3.603 PMC2995496

[32] Chanda D, Li T, Song K-H, Kim Y-H, Sim J, Lee CH, et al. Hepatocyte growth factor family negatively regulates hepatic gluconeogenesis via induction of orphan nuclear receptor small heterodimer partner in primary hepatocytes. J Biol Chem. 2009;284:28510–21. 10.1074/jbc.M109.022244. 19720831 10.1074/jbc.M109.022244 PMC2781394

[33] Anitha K, Dua K, Chellappan DK, Gupta G, Singh SK, Lakshmi SM, et al. HGF/c-MET: a potential target for the treatment of various cancers. CEI. 2023;19:71–80. 10.2174/1573408019666230227101036.

[34] Yap TA, Sandhu SK, Alam SM, Bono J. HGF/c-MET targeted therapeutics: novel strategies for cancer medicine. Curr Drug Targets. 2011;12:2045–58. 10.2174/138945011798829348. 21777195 10.2174/138945011798829348

[35] Dandona P, Aljada A, Bandyopadhyay A. Inflammation: the link between insulin resistance, obesity and diabetes. Trends Immunol. 2004;25:4–7. 10.1016/J.IT.2003.10.013. 14698276 10.1016/j.it.2003.10.013

[36] Boden G. Obesity, insulin resistance and free fatty acids. Curr Opin Endocrinol Diabetes Obes. 2011;18:139–43. 10.1097/MED.0b013e3283444b09. 21297467 10.1097/MED.0b013e3283444b09 PMC3169796

[37] Boden G, Free Fatty Acids A, Major Link. Between Obesity, insulin Resistance, Inflammation, and atherosclerotic vascular disease. In: Fonseca VA, editor. Cardiovascular endocrinology. Totowa, NJ: Humana; 2009. pp. 61–70. 10.1007/978-1-59745-141-3_4.

[38] Sarnings W, Aman AM, Rasyid H, Bakri S, Sanusi H, As Daud N, et al. Obesity measurement index is associated with hemoglobin A1c level in young adult without diabetes: a single-center cross-sectional study. J Endocrinol Metab. 2022;12:140–5. 10.14740/jem823.

[39] Bower JK, Meadows RJ, Foster MC, Foraker RE, Shoben AB. The association of percent body fat and lean mass with HbA1c in US adults. J Endocr Soc. 2017;1:600–8. 10.1210/js.2017-00046. 29264513 10.1210/js.2017-00046 PMC5686694

[40] Jørgensen ME, Borch-Johnsen K, Stolk R, Bjerregaard P. Fat distribution and glucose intolerance among Greenland Inuit. Diabetes Care. 2013;36:2988–94. 10.2337/dc12-2703. 23656981 10.2337/dc12-2703 PMC3781528

[41] Borel AL, Nazare JA, Smith J, Aschner P, Barter P, van Gaal L, et al. Visceral, subcutaneous abdominal adiposity and liver fat content distribution in normal glucose tolerance, impaired fasting glucose and/or impaired glucose tolerance. Int J Obes. 2015;39:495–501. 10.1038/ijo.2014.163. 10.1038/ijo.2014.163 25179244

[42] Newell-Morris LL, Treder RP, Shuman WP, Fujimoto WY. Fatness, fat distribution, and glucose tolerance in second-generation Japanese-American (Nisei) men. Am J Clin Nutr. 1989;50:9–18. 10.1093/AJCN/50.1.9. 2750700 10.1093/ajcn/50.1.9

[43] Feskens EJ, Kromhout D. Effects of body fat and its development over a ten-year period on glucose tolerance in euglycaemic men: the Zutphen study. Int J Epidemiol. 1989;18:368–73. 10.1093/IJE/18.2.368. 2767850 10.1093/ije/18.2.368

[44] Winner D, Norton L, Kanat M, Arya R, Fourcaudot M, Hansis-Diarte A, et al. Strong association between insulin-mediated glucose uptake and the 2-hour, not the fasting plasma glucose concentration, in the normal glucose tolerance range. J Clin Endocrinol Metab. 2014;99:3444–9. 10.1210/jc.2013-2886. 24796924 10.1210/jc.2013-2886 PMC4154101

[45] Yeckel CW, Taksali SE, Dziura J, Weiss R, Burgert TS, Sherwin RS, et al. The normal glucose tolerance continuum in obese youth: evidence for impairment in beta-cell function independent of insulin resistance. J Clin Endocrinol Metab. 2005;90:747–54. 10.1210/JC.2004-1258. 15522932 10.1210/jc.2004-1258

[46] Weigensberg MJ, Cruz ML, Goran MI. Association between insulin sensitivity and post-glucose challenge plasma insulin values in overweight Latino youth. Diabetes Care. 2003;26:2094–9. 10.2337/DIACARE.26.7.2094. 12832319 10.2337/diacare.26.7.2094

[47] Zhang Y, Xia M, Jin K, Wang S, Wei H, Fan C, et al. Function of the c-Met receptor tyrosine kinase in carcinogenesis and associated therapeutic opportunities. Mol Cancer. 2018;17:45. 10.1186/s12943-018-0796-y. 29455668 10.1186/s12943-018-0796-y PMC5817860

[48] Dehghan M, Merchant AT. Is bioelectrical impedance accurate for use in large epidemiological studies? Nutr J. 2008;7:26. 10.1186/1475-2891-7-26. 18778488 10.1186/1475-2891-7-26 PMC2543039

