# Supplementary material for: The role of hepatocyte growth factor in the relationship between body fat distribution and plasma markers of glucose metabolism: a cross-sectional study
Source: Diabetol Metab Syndr. 2026 Jan 27;18:68. doi: 10.1186/s13098-026-02096-1 (PMC12918471; doi:10.1186/s13098-026-02096-1)
Supplement: Supplementary file 1 — Supplementary Material 1 [file 13098_2026_2096_MOESM1_ESM.docx]

**Supplementary material**

**The role of hepatocyte growth factor in the relationships between body fat distribution and plasma markers of glucose metabolism**

Katarina Zakic, Dennis Freuer, Jakob Linseisen, Christa Meisinger

**
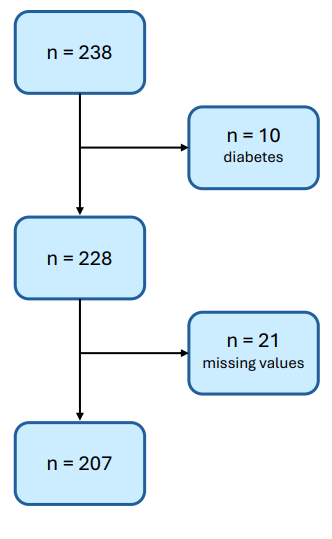
**

**Figure S1:** Flowchart showing the selection process for participants included in the regression analyses.


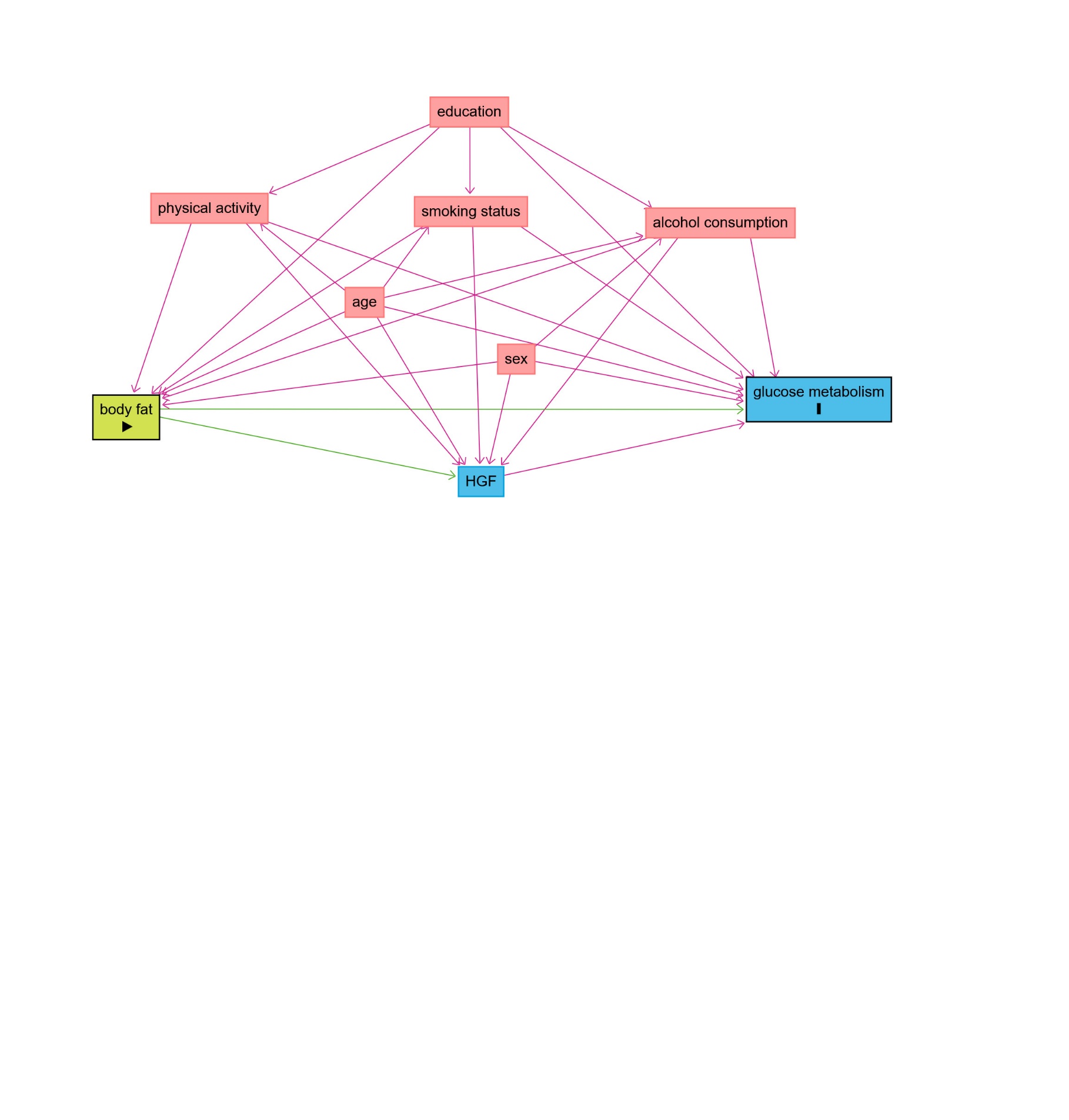


**Figure S2:** Directed acyclic graph (DAG) used for confounder selection. Red rectangles represent potential confounding factors of the body fat - glucose metabolism association. The blue square at the bottom represents a mediator.

**Table S3:** Results from Mediation analyses

|  | **X->M** | | | | **M->Y** | | | |  | **Indirect Effect (X->M->Y)** | | **Interaction** |  |
| --- | --- | --- | --- | --- | --- | --- | --- | --- | --- | --- | --- | --- | --- |
| **X** | **Beta** | **cil** | **ciu** | **P** | **Beta** | **cil** | **ciu** | **P** | **Beta** | **Boot cil** | **Boot ciu** | **P(X*M)** | **Mediation (%)** |
| Fasting plasma glucose levels (mg/dL) | | | | | | | | | | | | | |
| VAT | 26.30 | 7.09 | 45.51 | 0.008 | 0.00 | -0.01 | 0.01 | 0.938 | 0.01 | -0.50 | 0.38 | 0.138 | no mediation |
| AFMV | 18.54 | 2.59 | 34.50 | 0.023 | 0.00 | -0.01 | 0.01 | 0.832 | 0.03 | -0.30 | 0.30 | 0.402 | no mediation |
| RFMV | 18.02 | 0.52 | 35.53 | 0.044 | 0.00 | -0.01 | 0.02 | 0.696 | 0.05 | -0.21 | 0.35 | 0.986 | no mediation |
| BMI | 23.44 | 7.28 | 39.60 | 0.005 | 0.00 | -0.01 | 0.01 | 0.884 | -0.02 | -0.38 | 0.28 | 0.336 | no mediation |
| WC | 28.04 | 10.42 | 45.66 | 0.002 | 0.00 | -0.02 | 0.01 | 0.806 | -0.05 | -0.51 | 0.32 | 0.092 | no mediation |
| WHR | 32.84 | 12.38 | 53.29 | 0.002 | 0.00 | -0.01 | 0.02 | 0.772 | 0.07 | -0.41 | 0.57 | 0.147 | no mediation |
| Two-hour plasma glucose levels (mg/dL) | | | | | | | | | | | | | |
| VAT | 26.14 | 6.45 | 45.83 | 0.010 | 0.06 | 0.01 | 0.10 | 0.010 | 1.48 | -0.96 | 3.68 | 0.172 | 8.3 |
| AFMV | 21.18 | 4.59 | 37.77 | 0.013 | 0.06 | 0.02 | 0.10 | 0.008 | 1.26 | 0.01 | 3.20 | 0.417 | 9.1 |
| RFMV | 18.91 | 0.80 | 37.02 | 0.041 | 0.06 | 0.02 | 0.10 | 0.005 | 1.15 | -0.02 | 3.02 | 0.893 | 7.0 |
| BMI | 26.62 | 9.82 | 43.43 | 0.002 | 0.05 | 0.01 | 0.09 | 0.019 | 1.37 | 0.06 | 3.34 | 0.431 | 8.6 |
| WC | 29.71 | 11.43 | 47.99 | 0.002 | 0.05 | 0.01 | 0.09 | 0.024 | 1.46 | 0.07 | 3.54 | 0.350 | 8.1 |
| WHR | 33.02 | 11.04 | 55.01 | 0.003 | 0.05 | 0.01 | 0.10 | 0.019 | 1.71 | 0.13 | 4.30 | 0.057 | 8.1 |
| HbA1c levels (mmol/mol) | | | | | | | | | | | | | |
| VAT | 26.09 | 6.98 | 45.21 | 0.008 | 0.00 | -0.01 | 0.00 | 0.739 | -0.02 | -0.22 | 0.16 | 0.399 | no mediation |
| AFMV | 17.68 | 1.84 | 33.52 | 0.029 | 0.00 | -0.01 | 0.01 | 0.861 | -0.01 | -0.13 | 0.12 | 0.439 | no mediation |
| RFMV | 17.42 | 0.07 | 34.78 | 0.049 | 0.00 | -0.01 | 0.01 | 0.861 | -0.01 | -0.13 | 0.11 | 0.458 | no mediation |
| BMI | 22.44 | 6.39 | 38.49 | 0.006 | 0.00 | -0.01 | 0.01 | 0.742 | -0.02 | -0.17 | 0.12 | 0.386 | no mediation |
| WC | 27.11 | 9.58 | 44.65 | 0.003 | 0.83 | 0.16 | 1.51 | 0.015 | -0.03 | -0.18 | 0.14 | 0.178 | no mediation |
| WHR | 32.37 | 12.05 | 52.69 | 0.002 | 0.00 | -0.01 | 0.01 | 0.719 | -0.03 | -0.21 | 0.17 | 0.069 | no mediation |

X represents obesity measures, M the parameters for glucose metabolism, and Y the mediator HGF.
